# Activation of the hypnozoite: a part of *Plasmodium vivax *life cycle and survival

**DOI:** 10.1186/1475-2875-10-90

**Published:** 2011-04-16

**Authors:** Lena Hulden, Larry Hulden

**Affiliations:** 1 Department of Agricultural Sciences, Faculty of Agriculture and Forestry, P.O. Box 27, FI-00014 University of Helsinki, Finland; 2 Museum of Natural History, P.O. Box 17, FI-00014, University of Helsinki, Finland

## Abstract

**Background:**

*Plasmodium vivax *is the most widespread malaria parasite. It has a dormant stage in the human liver, which makes it difficult to eradicate. It is proposed that a relapse of vivax malaria, besides being genetically determined by the specific strain, is induced by the bites of uninfected vectors.

**Presentation of the hypothesis:**

The dormant stage maximizes the possibility for the parasite to reach the vector for sexual reproduction. The advantage would increase if the parasite was able to detect the presence of a new generation of vectors. The sporozoites function both in the vector and in the human hosts. They invade the cells of the salivary gland in the vector and the hepatocytes in the human. Some of the sporozoites develop into hypnozoites in the human liver. It is suggested that the hypnozoite activates when it recognizes the same *Anopheles *specific protein, which it had previously recognized as a sporozoite to invade the salivary gland in the vector. Another possibility is that the hypnozoite activates upon the bodily reaction by the human on a bite by an *Anopheles *female.

**Testing the hypothesis:**

The connection between the relapse and a new generation of vectors can be documented by simultaneous monitoring of both parasitaemia in humans and the presence of uninfective/infective vectors in the same area with seasonal malaria transmission. Experimental studies are needed to find the saliva components, which trigger the relapse. Although *P. cynomolgi *in monkeys also has hypnozoites and relapses, testing with monkeys might be problematical. These live in a reasonably stable tropical environment where relapses cannot easily be linked to vectors. The importance of the trigger increases in unpredictable variations in the vector season.

**Implications of the hypothesis:**

Artificial triggering of hypnozoites would make the medication more effective and resistance against a protein that the parasite itself uses during its life cycle would not develop. In areas with seasonal vivax malaria it could be used locally for eradication.

## Background

*Plasmodium vivax *is the most common human malaria species outside Africa with 2.6 billion people at risk in South Asia, Southeast Asia, South and Central America [1]. It is the most widespread malaria parasite and was, until the middle of the 20th century present in almost the whole inhabited world with presumably the exception of West and Central Africa [2]. *Plasmodium vivax *has a dormant stage in the human liver. After the sporozoites enter the hepatocytes not all will develop into schizonts, but some remains as hypnozoites [3]. The hypnozoites can remain dormant for months, or even years and the mechanism behind the development into dormancy and the activation is not known. The hypnozoites are insensitive to atovaquone-proguanil, which is active against liver stage schizonts and to chloroquine [4]. Relapses indicating a dormant stage occur also in *Plasmodium ovale *and in the simian *Plasmodium cynomolgi, Plasmodium fieldi, Plasmodium simiovale *and *Plasmodium schwetzi *infections [5-7]. A dormant stage in *P. ovale *has recently been questioned [8]. *Plasmodium falciparum *and *Plasmodium malariae *do not have a dormant liver stage. Instead, the gametocytes of *P. falciparum *can survive in the blood for months [9] and *P. malariae *can cause long time chronic infections, which reoccur decades after the initial exposure [10].

The length of the dormancy varies between different strains of *P. vivax*. It is only 17 days in the Chesson strain from New Guinea [4], but in the eastern parts of Finland relapses could be statistically detected at least nine years after the primary infection [11]. The strain from Eastern Finland is now extinct, but it was probably close to the *hibernans *strain. The latter was isolated from north of Moscow [12]. A long dormancy is also known from tropical areas. A length of four years in a patient from Cameroon has been reported in Italy [13].

The proportion of how many hypnozoites are produced from the sporozoites injected with one vector bite is unknown. It has been suggested that more hypnozoites are produced when the infected mosquitoes are housed at a cold temperature [14]. The variation in the dormancy period of the hypnozoites is a polymorphic character in *P. vivax*, and the variation in the relapse time can be explained as a result of regional and seasonal variation in the mosquito vector. Unpredictable seasonality or unpredictable vector populations will select for a character that produces hypnozoites with a long dormancy. It is unlikely that a fixed length of dormancy alone would have ensured the survival of *P. vivax *through evolutionary time scales. It is probable that the parasite also has some means of finding the vector. Ability for the hypnozoites to detect the presence of a new generation of vectors would explain the geographical success of *P. vivax*. Colder regions in the temperate zone correlate with the increase of long dormancy period of vivax malaria. A long dormancy in the tropics is less pronounced because the relapse is easily hidden by continuous presence of mosquitoes.

A relapse of vivax malaria, besides being genetically determined by the specific strain, could also be induced by the bites of uninfected vectors. This was statistically indicated in the long time dataset from Finland [11]. The Finnish domestic vector, *Anopheles messeae*, has a strong seasonality. The females hibernate as adults through winter and lay eggs in the spring. The development of the larvae takes place in summer in permanent water, which freezes during the winter. Monitoring of mosquitoes with the commercial MosquitoMagnet has been done by the authors from May to October 2008-2010 both in the South and in the North of Finland. No adult *Anopheles *females were flying between the beginning/middle of June and late July/August. *Anopheles *were also absent in a study of mosquitoes attracted to man in Finland in the middle of summer [15]. When the next generation of adult hatches in late July/August, it is already too cold for the sporogony. The late summer/early autumn malaria top in the Finnish material had, therefore, to be relapses and they showed a distinct connection with the vector phenology [11].

The same pattern can be found from British material. Analyses of domestic relapses of imported *P. vivax *cases showed an adaption to the phenology of local vectors. On return to the UK, clinical malaria tended to develop in patients returning to Birmingham from Northern Pakistan during the summer months irrespective when the patients had got the original infection [16]. It was suggested that the cold winter months somehow "programmed" the parasites to correlate with the appearance of the biting mosquitoes. A comparison with the phenology from Finland shows, however, that this is not the case. There the number of malaria cases went down during the warm summer months, when the vectors only were present as larvae. A parsimonious explanation for both the Finnish and the British material is that the hypnozoites react on the local phenology of the vectors.

Other British studies show the same adaptation to local seasonality. A study made in Glasgow showed that the onset of imported vivax malaria became confined to and delayed to the British summer months irrespective of when the infection was contracted [17]. A similar larger study was done in London. The majority of the patients had got the infection in Punjab, India and some cases had been contracted in Africa and the Middle East. There was a great variation in the incubation periods and clinical attacks of malaria were almost absent during the winter months [18].

Unfortunately there is no monitoring of the presence of *Anopheles *females indoor in British suburban areas. A study made in Helsinki during the malaria epidemic 1901-1902 showed that active *Anopheles *females could easily be found in apartments in the middle of the city [19,20]. *Anopheles messeae *was abundant in the mosquito material collected by the authors with a trap at the university campus in Helsinki 2008-2009.

A case that describes simultaneous occurrence of relapses in two persons living together has also been described from Canada, where a volunteer couple had got infected with both *P. falciparum *and *P. vivax *in Papua New Guinea. After a holiday at Lake Winnipeg in August both the husband and the wife got a relapse within one week apart. The authors suggested that the hypnozoites were programmed genetically to be activated at defined intervals or that they reacted on outside stimuli as cold or stress [21]. It should be noted that also in this case the relapses occurred during the local *Anopheles *season.

### Hypnozoites triggered by uninfected mosquitoes?

The biology behind the dormancy is unknown. The investment of some of the sporozoites into hypnozoites gives the parasite several advantages. It will maximize the possibility for the parasite to reach the vector for sexual reproduction because the human host will be infective on more than one occasion. A strain-dependant minimum time during which the hypnozoite remains inactive before it can be activated may also be assumed.

The advantage increases considerable if the activation of the hypnozoites does not happen at random or is only genetically "programmed", but are triggered by the parasite. Activation during the vector-free season would be disastrous in areas with seasonal vectors. Instead, it is proposed that the relapses are triggered by the bites of uninfected vectors. In the long time series from Finland, relapses are shown to coincide with the seasonality of the main vector [11]. The adult mosquitoes hatch during the end of the summer, and after mating the males die and the females seek a hibernation place. During this time there were no infective mosquitoes present but the number of malaria cases increased. Blood feeding is not a prerequisite, but 15% of the females still take blood before hibernation [22].

*Plasmodium vivax *has probably evolved in Southeast Asia between 217,000 and 304,000 years ago when humans lived in small nomadic groups [23]. A human host brought new challenges for the parasite. The humans had bigger territories than the tree living monkeys. For *P. vivax*, it was necessary to have access to both the human and the vector. The human group would not necessarily always stay at a place long enough for the sporogony to be completed. The groups were small, which affected the number of people that an infective vector could bite. Even though the seasonality of the vector is less pronounced in the tropics than in Northern areas, there still is a difference between the dry and rainy season. Vivax malaria was probably a rare disease in the Palaeolithic society with hunters and gatherers. The dormant stage with an ability to recognize the new generation of vectors would have insured the survival of the parasite during periods without vectors. Triggered relapses would efficiently infect the vectors and insure the transmission to new human hosts.

The development of agriculture brought human in a much closer contact with the *Anopheles *mosquitoes than before. Larvae of *Anopheles *are mostly found in water bodies like permanent and semi-permanent pools, rice fields, margins of lakes and rivers with reduced water flow, and puddles or ditches with a growth of aquatic vegetation [24]. Only when humans built permanent settlements near the optimal breeding places, malaria started to be an essential health problem.

The Finnish malaria data showed a correlation between the newly hatched, uninfective *Anopheles *females and the late summer/early autumn top of malaria cases. The earlier phenology of the other man-biting species did not correlate with malaria cases [11]. An ability to recognize the presence of a suitable vector would also make the dispersal of *P. vivax *very effective. This was indicated during the gold rush in Klondyke. Thousands of prospectors rushed to Dawson city in 1897 and 1898 where they suffered severely from malaria [25]. Several of the newcomers carried hypnozoites and the parasite adapted easily to the local common vector species, *Anopheles earlei *[26]. Malaria ceased to be a problem with no relapses when the prospectors moved from Klondyke further westwards to Nome on the tundra in Alaska, where there were no local *Anopheles *species present.

The *Anopheles*-bite triggered relapse would, beside to ensure the survival of the parasite through a vector free season, allow effective dispersal to new areas and provide better possibilities for the parasite to reach a vector. The control of the activation of the hypnozoites might also increase the possibility for *P. vivax *to have meiotic recombination in the mosquitoes. A human could then have several primary infections without relapses. With every new infection some of the sporozoites would remain as hypnozoites in the liver. When the hypnozoites are activated by the bites of uninfected vectors there would be a synchronous development of hypnozoites from different strains [27]. The mechanism would explain the considerable genetic diversity in *P. vivax*, which has been emphasized in several studies [28]. It has also been showed that the genotypes of the *P. vivax *population are often different from the parasite that caused the primary infection [29]. A simultaneous relapse of hypnozoites from different infections would also explain the genetically different parasites present in multiple infections that have been shown in studies from India [30,31].

The success of malaria transmission depends on the sporozoites, which can function in both the vector and the human host. They develop in the oocysts and are released into the haemocoelic cavity. The sporozoites are then transported by the circulation [32] of the haemolymph or they follow the continuum of the basal lamina of the midgut until they come to the surface of the salivary gland [33]. Only 19% of the sporozoites will reach the salivary gland [34]. The sporozoite invades only the salivary gland, not any of all the other cells and tissues of the mosquito, which means that they are able to differentiate between them. The invasion of the salivary gland probably requires species-specific receptor-ligand interactions [32,35]. The trans-membrane surface protein TRAP (thrombospondin-related anonymous protein) mediates cell invasion of the sporozoite and has an important role for the locomotion. Another transmembrane protein of the sporozoite, an apical membrane antigen/erythrocyte binding-like protein (MAEBL) mediates salivary gland recognition and adhesion [36].

The sporozoite invasion of *P. falciparum *into the salivary glands of *Anopheles gambiae *and *Anopheles stephensi *has been analysed by Ghosh *et al *[37]. The twelve-amino-acid peptide SM1 binds tightly to the salivary gland and inhibits the sporozoite invasion and *Anopheles *salivary protein saglin is a receptor for SM1 [37]. The peptide is a mimotope of the sporozoite TRAP and saglin/TRAP interaction seems to be important for the salivary gland invasion [37]. The infective vector then injects the sporozoite into the human host. The behaviour of the sporozoites changes depending on where they are and the migratory activity was increased in non-hepatic cells compared to hepatocytes [38].

The biological determinant of the sporozoites to develop into either active schizonts or dormant hypnozoites is unknown [3]. The simplest hypothesis would be that not all sporozoites enter the hepatocytes at the same time. It has been assumed that the sporozoites usually leave the injection site within minutes of the bite. Experiments with mice have showed that the sporozoites can remain infective for a long period in the mammalian host and that some sporozoites begin to develop in the draining lymph node [38]. Perhaps the development of schizonts in the hepatocytes also deters the development of other sporozoites. The late-comers will instead survive as dormant hypnozoites. The ability for a *Plasmodium *to react on the vicinity of another parasite is known, but not in the liver stages. The merozoites of *P. falciparum *avoid penetrating cells, which are already occupied [39].

When the hypnozoite lies dormant in the human hepatocyte it has already during its life cycle recognized the mosquito salivary gland. The vector injects saliva into the host during feeding. The hypnozoite could react with a protein in the vector saliva, which is specific for the *Anopheles *genus. Saglin could be one of the candidates. Another possibility is that the hypnozoite activates upon the bodily reaction by the human on a bite by an *Anopheles *female. A study with sera from malaria patients, healthy villagers and people from non-malarious regions in Thailand showed that the malaria patients reacted against mosquito antigens. They had anti-*Anopheles *salivary protein antibodies that were *Anopheles*-specific. There were also indications that the antibodies were specific for the *Anopheles *species [40]. Possibly both a human reaction and the protein from the vector saliva are needed. More research is needed because experiments with intravenous injections of sporozoites of *P. cynomolgi *dissected from salivary glands to monkeys also resulted in relapses [5].

### Testing the hypothesis

The analysis of the Finnish material was based on historical data without the use of quinine or Peruvian bark. Although it was statistically tested, it should be compared with modern material in which the malaria cases are diagnosed by blood smear. The British material should also be reanalysed and compared with the local vector distribution and phenology. The problem with the analyses of imported cases is, however, the possible dependency on the original vector. The triggered relapse might be caused by a combination of several components in the saliva and a species dependent human reaction against the bite. It is also possible that the hypnozoites will only be activated if the human carrier has antibodies to the potential vector species. If a human carrier moves to an area with different vector species, the hypnozoites would in that case only react after one mosquito season when the carrier has acquired antibodies against the possible new vector.

More statistics of malaria cases and data from vector monitoring should be collected. A thorough follow-up of vivax malaria cases together with vector monitoring in an area with seasonal malaria transmission should, therefore, be done. The infectivity of the potential vectors should be determined and their timing compared to the malaria cases. The connection between the relapse and a new generation of vectors can be documented by simultaneous monitoring of both parasitaemia in humans and the presence of uninfective/infective vectors in the same area.

Experimental studies are needed to find the recognizable saliva components, which triggers the relapse. Testing with *P. cynomolgi *on monkeys might be problematical. It infects rhesus macaques in Southeast Asia and is closely related to *P. vivax *[41]. The clinical infection of both parasites is very similar. *P. cynomolgi *also has the dormant liver stage with hypnozoites that causes the relapsing blood stage infection [40]. The evolution of *P. cynomolgi *is closely related to monkeys, which are highly tropical animals and live in a reasonably stable environment. Their territory is relatively small and the vector will usually find its primate host. The vector would, therefore, from the parasite's point of view, always be easy to find. The circumstances for *P. vivax *are almost the opposite. It has been worldwide successful because it has been able to adapt to an environment with a highly irregular presence of vectors.

### Implications of the hypothesis

The determination of a component in the vector saliva, which induces or triggers the relapse of vivax malaria, would change the medication, since it is easier to treat the parasite in the bloodstream than the hypnozoites in the liver. Primaquine resistance has already appeared and will be more common in the future. Resistance against a protein that the parasite itself uses for activation could not develop. If the hypnozoites could be artificially activated the possibilities for efficient medication would increase. Drug resistance, which is a growing problem, is a result of a parasite adapting to a change in its environment. It will, however develop slower with controlled relapses, simply because the number of inadequately treated relapses originating from one infection would decrease.

Activation of the hypnozoites is the key for the survival of *P. vivax *in areas with seasonal vectors. It could, however, also be used as a way of effective vivax eradication on a regional scale. By activating the hypnozoites out of mosquito season and treating the patients, the number of infective vectors would be reduced during the next malaria season; in combination with vector control indoors and bed nets, eradication could be achieved faster than only with traditional means.

## Competing interests

The authors declare that they have no competing interests.

## Authors' contributions

LeH drafted the manuscript. LaH argued and both authors finalized the manuscript together. Both authors read the final manuscript and approved the submission.
